# Desmoplastic myxoid tumor, SMARCB1-mutant: a case with late recurrence after long-term follow-up

**DOI:** 10.17879/freeneuropathology-2026-9701

**Published:** 2026-07-06

**Authors:** Arnault Tauziède-Espariat, Mathilde Duchesne, Aymeric Rouchaud, Amandine Chabernaud Negrier, Romain Coudert, Joseph Benzakoun, David Guyon, Farah Sassi, Lauren Hasty, Alice Métais, Pascale Varlet

**Affiliations:** 1 Department of Neuropathology, GHU Paris-Psychiatrie et Neurosciences, Sainte-Anne Hospital, Paris, France; 2 Department of Pathology, Dupuytren University Hospital, Limoges, France; 3 Department of Radiology, Dupuytren University Hospital, Limoges, France; 4 Department of Neuroradiology, GHU Paris-Psychiatrie et Neurosciences, Sainte-Anne Hospital, Paris, France; 5 Department of Oncology, Gustave Roussy, Université Paris-Saclay, Villejuif, France

**Keywords:** Desmoplastic myxoid tumor, Pineal, SMARCB1, Ultrastructural analysis

## Letter

The desmoplastic myxoid tumor, *SMARCB1*-mutant (DMT) was introduced in the last World Health Organization (WHO) Classification of Tumors of the Central Nervous System (CNS) in the chapter on pineal tumors. According to essential diagnostic criteria, it is defined as a neoplasm characterized by: 1) desmoplasia and myxoid changes, 2) lack of histopathological signs of malignancy, and 3) loss of tumoral SMARCB1 expression. For unresolved cases, confirmation using methylation profiling analysis is required. Although all cases have been reported in the pineal region, curiously, the location is not included as an essential criterion of this new tumor type. Since the first description, only 14 cases have been described in the literature, and no cell of origin has been identified. Herein, we report a novel observation and perform an ultrastructural analysis to better characterize this tumor type.

**Figure 1: Initial radiological features of the tumor:**
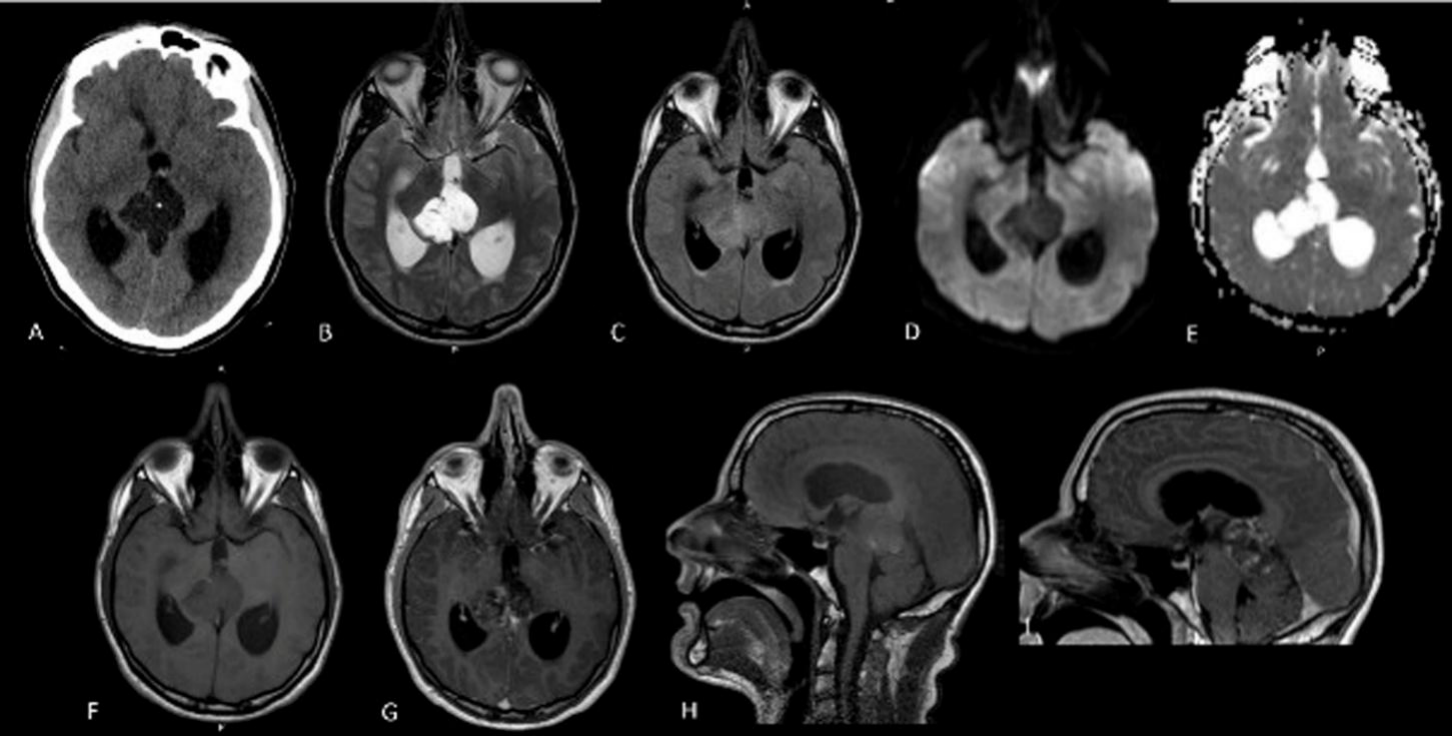
(**A**) Brain computed tomography (CT) images demonstrated a space-occupying lesion in the pineal region associated with obstructive hydrocephalus. The lesion was markedly hypodense with a pseudo-cystic appearance and contained a 3-mm macrocalcification. A small amount of intraventricular hemorrhage was visible in its anterior portion within the third ventricle. (**B–I**) Magnetic resonance imaging (MRI) showed the lesion to be lobulated and markedly hyperintense on T2-weighted imaging (**B**), with a pseudo-fluid signal intensity and intermediate signal intensity on T2 fluid-attenuated inversion recovery (FLAIR) imaging (**C**). There was no diffusion restriction, with low signal intensity on diffusion-weighted imaging (DWI) (**D**) and high signal intensity on apparent diffusion coefficient (ADC) maps (**E**). The lesion was predominantly isointense on T1-weighted images (**F–H**), with a few spontaneously T1-hyperintense areas suggestive of hemorrhagic or proteinaceous content. Partial heterogeneous enhancement was observed after contrast administration (**G–I**).

**Figure 2: Histopathological features of the tumor:**
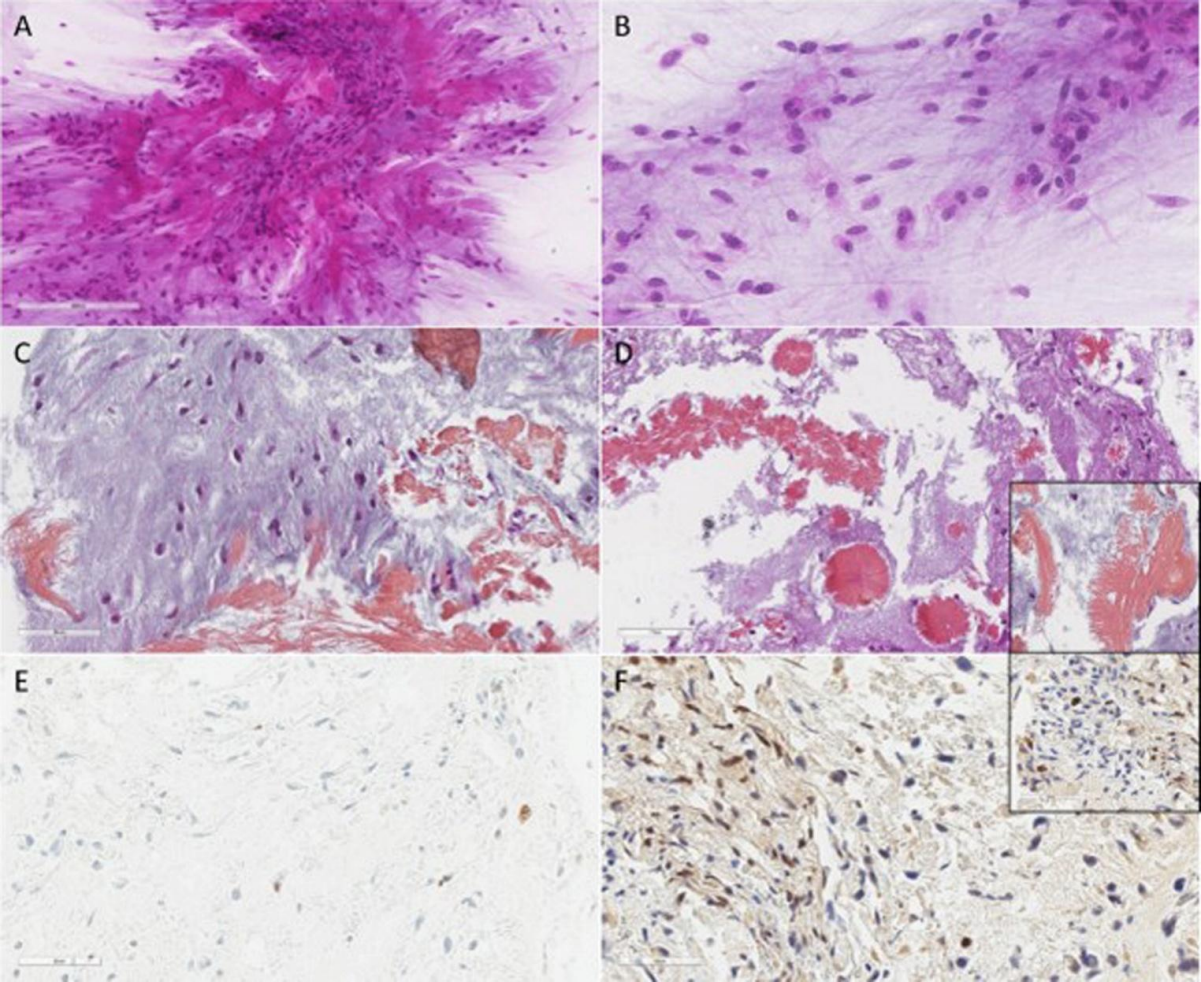
(**A–B**) The microscopic examination of the smear revealed the presence of a poorly cellular tumor composed of spindle cells, in a myxoid background with numerous hyalinized vessels (May-Grünwald Giemsa staining, magnification x400). (**C–D**) After fixation, the tumor was composed of thin cords of spindle cells presenting oval nuclei without atypia arranged in a myxoid stroma with numerous collagen fibers (Hemalun Phloxin Saffron staining, magnification x400) and crystalloids (Hemalun Phloxin Saffron staining, magnification x300, and insert x400). (**E**) MIB1 labeling was very low (magnification x400). **(F**) Loss of INI1/SMARCB1 immunoexpression in tumor cells (magnification x400 and insert x800). Scale bars represent 50 μm (**A–C** and **E–F**) and 70 μm (**D**).

**Figure 3: Radiological features of the recurrence eleven years after the initial surgery:**
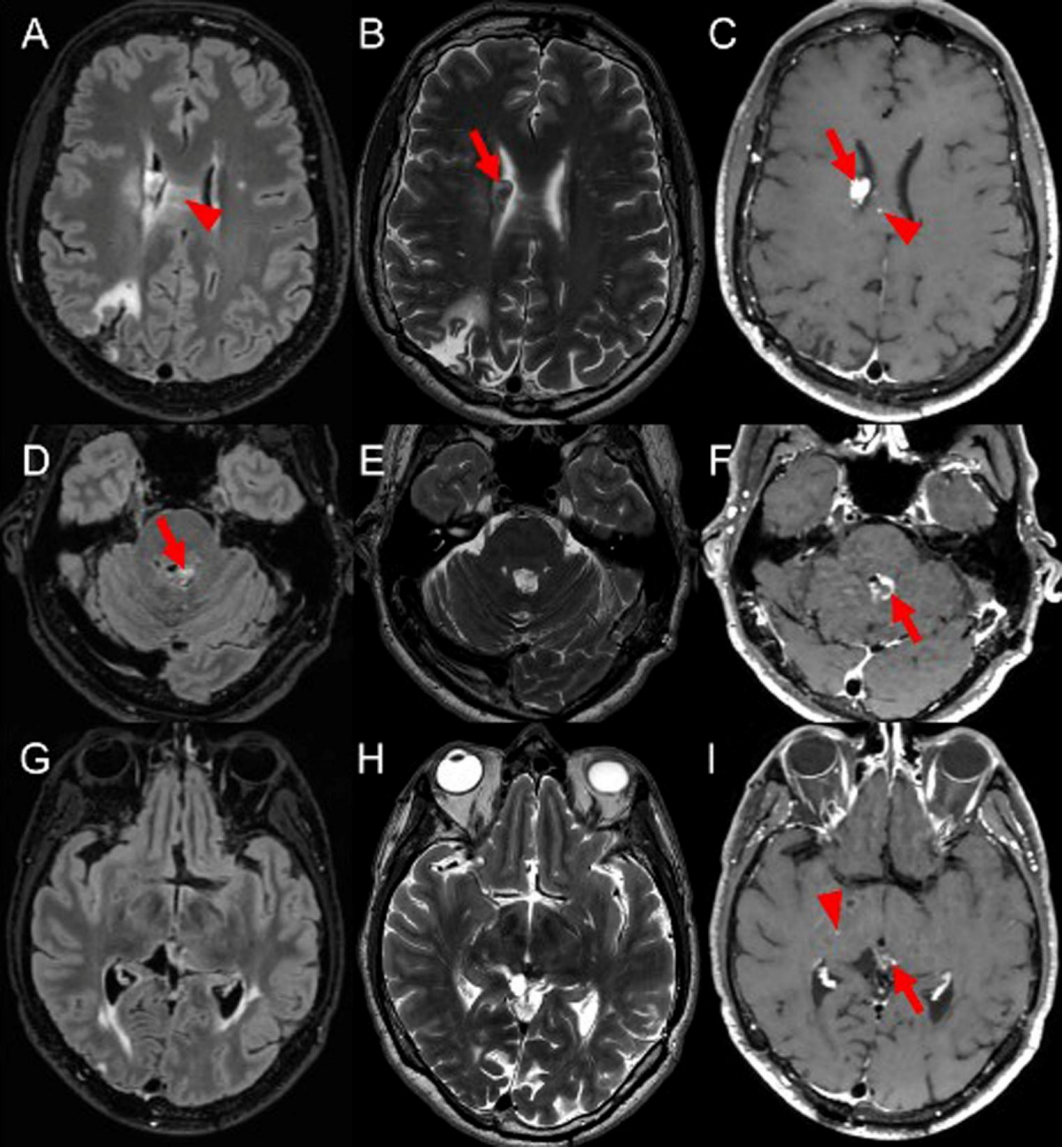
FLAIR, T2 and contrast-enhanced T1 sequences are respectively presented in the first, second, and third columns. (**A–C**) Axial slices centered on the right ventricle display a subependymal nodule (arrow in **B**) with intense enhancement (arrow in** C**), suggesting a recurrence. The presence of a focal enhancement of the corpus callosum (arrowhead in **C**) and FLAIR hyperintensities of the corpus callosum (arrowhead in **A**) are also noted, suggestive of tumoral infiltration. (**D–F**) Axial slices centered on the fourth ventricle display a subependymal nodule (arrow in **D**) with intense peripheral enhancement (arrow in** F**), suggesting a recurrence. (**G–I**) Axial slices centered on the pineal region display non-specific enhancements (arrow in **I**), as well as focal enhancement of the right thalamus (arrowhead in **I**) without any mass effect.

**Figure 4: Ultrastructural features of the tumor:**
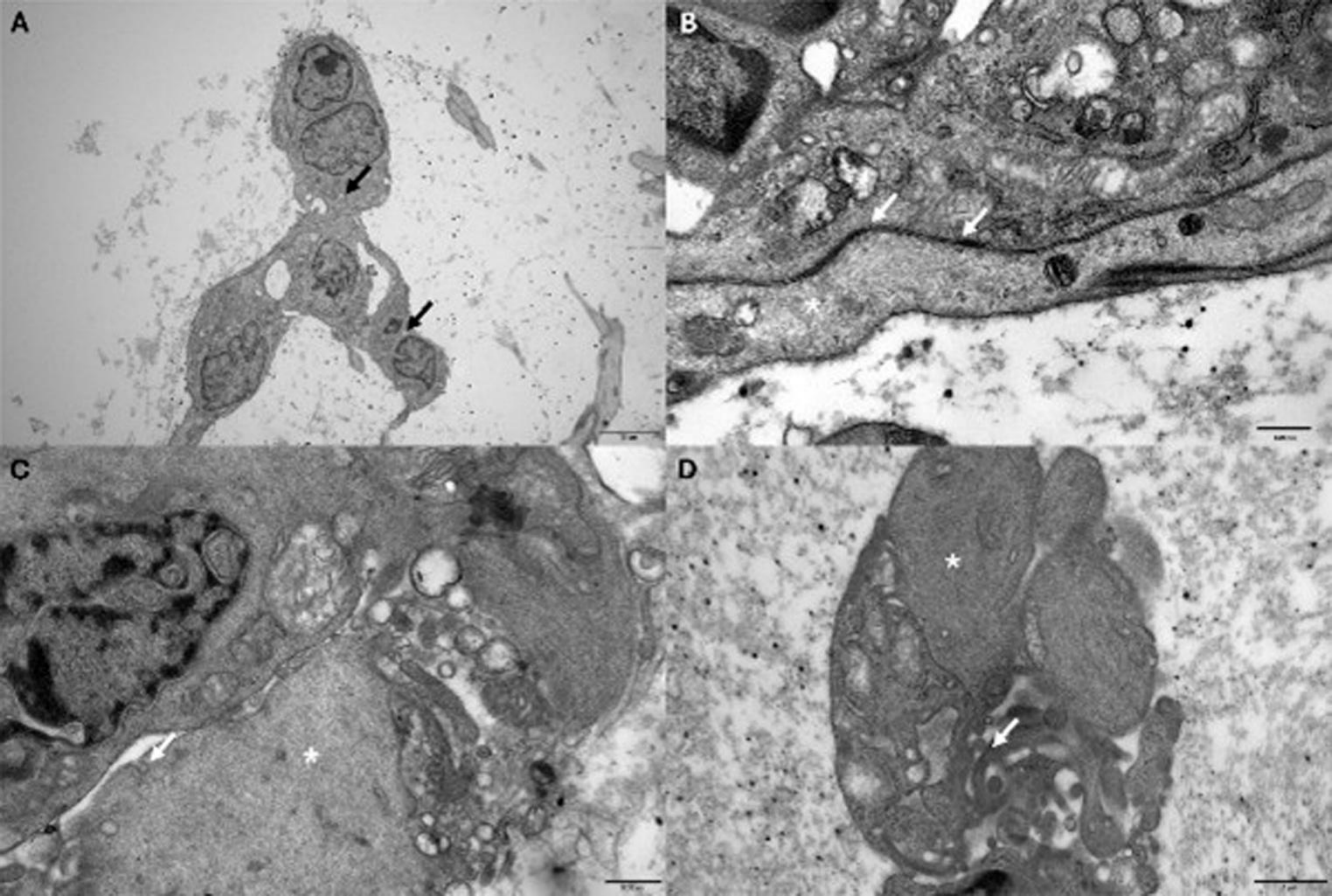
The ultrastructural examination of the tumor cells revealed large nuclei with prominent nucleoli (**A**), a scant cytoplasm containing small vacuoles (**A**, black arrow) and intermediate filaments (**B**–**D**, white asterisk). Several intercellular junctions are present (**C**, white arrow), as well as vesicular endocytosis (**C**, white arrow) and finger-like cytoplasmic extensions corresponding to microvilli (**D**, white arrow).

The current observation concerns a previously healthy 33-year-old man who presented with symptoms of intracranial hypertension. Brain computed tomography (CT) and magnetic resonance imaging (MRI) (**Figure 1A–I**) demonstrated a space-occupying lesion in the pineal region associated with obstructive hydrocephalus. The lesion was markedly hypodense with a pseudo-cystic appearance and contained a 3-mm macrocalcification. A small amount of intraventricular hemorrhage was visible in its anterior portion within the third ventricle. There was no other lesion in the CNS (including the cerebellum, the pineal gland, and leptomeninges) and no evidence of a developmental abnormality elsewhere in the brain. During the surgery, a fresh sample was analyzed, which showed the presence of a poorly cellular tumor composed of spindle cells, in a myxoid background with numerous hyalinized vessels (**Figure 2A–B**). After fixation, the definitive microscopic examination confirmed the preliminary results. The tumor was composed of thin cords of spindle cells, presenting oval nuclei without atypia or mitosis. The stroma was myxoid with numerous collagen fibers frequently arranged in amianthoid fibers and crystalloids (**Figure 2C–D**). There was no rhabdoid, epithelioid or meningothelial component, and there were no whorls or psammoma bodies. No necrosis was observed. Immunohistochemical analyses exhibited the expression of EMA by a subset of tumor cells, without immunoreactivity for GFAP, OLIG2, pan-cytokeratins, CD34, PS100, neurofilament, synaptophysin, smooth muscle actin or desmin. The MIB1 labeling index was low, less than 1 % (**Figure 2E**). The tumor was initially classified as a grade 1 metaplastic meningioma (myxoid variant), because the diagnosis was made before 2021. A partial resection of the lesion was first realized. The tumor recurred 8 months later, and a second resection was performed, followed by focal radiation therapy. The tumor residue was stable for 11 years, after which secondary locations appeared (a subependymal nodule and an infiltration of the corpus callosum) (**Figure 3A–I**). The patient is now treated by chemotherapy. A revised update of the initial diagnosis was recently performed. The tumor was SSTR2a negative by immunohistochemistry and showed a loss of INI1/SMARCB1 expression (**Figure 2F**). DNA-methylation profiling classified the sample as DMT (using the NIH classifier v2.0 with a calibrated score of 0.84, whereas it was not classified using the DKFZ classifier v12.8, as expected, given that DMT is not included in this last classifier). The copy number profile confirmed the presence of a *SMARCB1 *homozygous deletion. Ultrastructure analysis showed the tumor cells to be few in number, evolving in a loose matrix. They showed indented nuclei, extensive *zonulae adherens* junctions, vesicular endocytosis, finger-like projections and intermediate filaments (**Figure 4**).

 DMT affects young adults (median age: 33 years-old, from 15 to 61 years-old) with a female-to-male ratio of 2 **(1) **(see **Table 1** for a summary of the literature review). Despite their exclusive location in the pineal region, no cell of origin has been identified to date. The histopathological features of the stroma (myxoid background with desmoplasia and collagen fibers) and the morpho-logy of tumor cells (spindle) may suggest a potential mesenchymal origin. Very few data are available concerning the immunohistochemical profile of this tumor type. The most frequent markers expressed are CD34, EMA and vimentin** (1,7)**, which are not specific. A potential multiphenotypic pattern has been suggested by Matsumura *et al. *with the coexpression of cytokeratins, GFAP, and synaptophysin **(3)**, and some other authors have reported an expression of smooth muscle actin **(1,7)** (the current presentation does not confirm these data). Given their morphology and immunophenotypical similarities, DMT and "metaplastic meningioma", "myxoid meningioma" or "chordoid meningioma" of the pineal region, may represent the same tumoral entity. As we know, the loss of expression of INI1/SMARCB1 is not typical of a specific origin (*SMARCB1* alterations may be present in sarcomas and carcinomas and low-grade diffusely infiltrative tumors (LGDIT)) and therefore does not in itself constitute evidence of the mesenchymal nature of this tumor. In order to further our understanding of the cellular origin of this tumor, we conducted an ultrastructural study. The ultrastructural features described here are similar to those described previously **(4)**. The cellular origin of this tumor type remains to be determined. The clinical behavior of this tumor type is variable, and no grading has been established so far. The particular location of this entity may explain the potential morbidity (3/12 patients dead of disease), rather than an intrinsic malignancy associated with the tumor. The current observation represents the longest follow-up period, in terms of years, following the initial diagnosis (more than ten years) and presents for the first time radiological evidence of dissemination. Further observations over a long period are needed in order to establish a definitive prognosis for this tumor type. 

**Table 1:** Clinical data of reported cases in the literature

**Author**	**Case**	**Age **	**Sex**	**Location**	**Treatment**	**Recurrence**	**PFS (months)**	**Status**	**OS (months)**
Thomas et al. (1)	1	53	F	pineal	PR	0	NA	Dead	0
Thomas et al. (1)	2	37	M	pineal	GTR	0	NA	Alive (stable disease)	9
Thomas et al. (1)	3	40	F	pineal	PR+CT+RT	1	NA	Dead	48
Thomas et al. (1)	4	32	F	pineal	GTR+RT	0	NA	Alive (stable disease)	57
Thomas et al. (1)	5	61	F	pineal	GTR	0	NA	Alive (stable disease)	86
Thomas et al. (1)	6	42	M	pineal	GTR	1	NA	Dead	51
Thomas et al. (1)	7	15	M	pineal	PR+RT	1	4	Alive (stable disease)	4
Wang et al. (1)	8	33	M	pineal	GTR	0	NA	Alive (without residual disease)	3
Matsumura et al. (1)	9	29	F	pineal	GTR	0	NA	Alive (without residual disease)	22
Manoranjan et al. (1)	10	24	F	pineal	STR+RT	0	NA	Alive (stable disease)	9
Zhou et al. (1)	11	50	F	pineal	GTR	0	NA	Alive (without residual disease)	6
Robinson et al. (1)	12	18	F	pineal	GTR	NA	NA	NA	NA
Goyal et al. (1)	13	22	F	pineal	NA	NA	NA	NA	NA
Goyal et al. (1)	14	45	F	pineal	NA	NA	NA	NA	NA

Abbreviations: CT: chemotherapy; F: female; GTR: gross total resection; M: male; NA: not available; OS: overall survival; PFS: progression-free survival; PR: partial resection; RT: radiation therapy; STR: subtotal resection.

To conclude, we present a novel observation of DMT, with a long follow-up and, for the first time, radiological evidence of dissemination.

## Ethics approval

This study was approved by the ethic board. This study was approved by the GHU Paris-Neuro-sciences Sainte-Anne’s local ethics committee (INDS: MR 1409210519).

## Authors’ contributions and consent for publication

ATE and MD participated in the conception, design, collection and assembly of data. MD and DG provided medical care for the patient. AR, ACN, RC, and JB conducted the radiological review. ATE, MD, FS, AM and PV conducted the neuropathological examinations. ATE drafted the manuscript and LH reviewed it. All authors reviewed the manuscript and approved the final version.

## Conflict of interest statement

The authors declare that they have no conflicts of interest directly related to the topic of this article.
